# The impact of a smartphone‐based cognitive aid on clinical performance during cardiac arrest simulations: A randomized controlled trial

**DOI:** 10.1002/aet2.10880

**Published:** 2023-06-07

**Authors:** Samuel L. Brophy, Michael R. McCue, Riley M. Reel, Tristan D. Jones, Roger D. Dias

**Affiliations:** ^1^ Faculty of Medicine University of British Columbia Vancouver British Columbia Canada; ^2^ Department of Emergency Medicine Vancouver Island Health Authority Victoria British Columbia Canada; ^3^ Department of Emergency Medicine Harvard Medical School Boston Massachusetts USA; ^4^ STRATUS Center for Medical Simulation Brigham and Women's Hospital Boston Massachusetts USA

## Abstract

**Objectives:**

In‐hospital cardiac arrests are common and associated with high mortality. Smartphone applications offer quick access to algorithms and timers but often lack real‐time guidance. This study assesses the impact of the Code Blue Leader application on the performance of providers leading cardiac arrest simulations.

**Methods:**

This open‐label randomized controlled trial included Advanced Cardiac Life Support (ACLS)–trained medical doctors (MD) and registered nurses (RN). Participants were randomized to lead the same ACLS simulation with or without the app. The primary outcome, “performance score,” was assessed by a trained rater using a validated ACLS scoring system. Secondary outcomes included percentage of critical actions performed, number of incorrect actions, and chest compression fraction (percentage of time spent performing chest compressions). A sample size of 30 participants was calculated to detect a difference of 20% at the 0.05 alpha level with 90% power.

**Results:**

Fifteen MDs and 15 RNs underwent stratified randomization. The median (interquartile range) performance score in the app group was 95.3% (93.0%–100.0%) compared to 81.4% (60.5%–88.4%) in the control group, demonstrating an effect size of *r* = 0.69 (*Z* = −3.78, *r* = 0.69, *p* = 0.0002). The percentage of critical actions performed in the app group was 100% (96.2%–100.0%) compared to 85.0% (74.1%–92.4%) in the control group. The number of incorrect actions performed in the app group was 1 (1) compared to 4 (3–5) in the control group. Chest compression fraction in the app group was 75.5% (73.0%–84.0%) compared to 75.0% (72.0%–85.0%) in the control group.

**Conclusions:**

The Code Blue Leader smartphone app significantly improved the performance of ACLS‐trained providers in cardiac arrest simulations.

## INTRODUCTION

### Background

The incidence of adult in‐hospital cardiac arrests (IHCA) in the United States is 10.16 per 1000 hospital admissions, which is approximately 292,000 per year.^1^, ^2^ Only 26.7% of these patients survive until discharge.^2^ The likelihood of return of spontaneous circulation (ROSC) from IHCA and survival to discharge is positively correlated with adherence to American Heart Association's Advanced Cardiovascular Life Support (ACLS) guidelines.^3^, ^4^, ^5^ More specifically, factors that are associated with decreased likelihood of ROSC or survival to discharge include delays in cardiopulmonary resuscitation (CPR) initiation, greater time to first defibrillation, incorrect voltage or timing of defibrillation, delays in rhythm identification, delays in medication administration or incorrect medications as per ACLS guidelines, and greater CPR interruptions, including those associated with endotracheal intubation.^3^, ^4^, ^5^

IHCA outcomes are also impacted by policies, including those that regulate resuscitation team design, mock codes, nursing empowerment, and frequency of IHCA case review.^6^, ^7^ Team design involves establishing resuscitation teams composed of highly trained health care professionals (HCPs) with defined responsibilities whose resuscitation role supersedes other clinical duties. This facilitates an organized response to IHCAs where all team members are familiar with each other and their roles.^6^ Nursing empowerment involves providing education and modifying policies to permit nurses to initiate interventions independently, such as rapid defibrillation and ACLS medication administration.^6^ While policy reform can improve IHCA outcomes to a degree,^6^ even experienced HCPs struggle to lead teams when cognitive overload occurs.^8^, ^9^ Therefore, other interventions may help to address this threat to patient care.^9^

### Importance

HCPs who lead IHCAs are often ACLS‐trained; however, the stress of these critical situations can exceed the cognitive capacity required to effectively follow ACLS algorithms.^9^ The Yerkes–Dodson law describes this phenomenon with their bell‐shaped model of arousal; while moderate levels of arousal yield optimal performance, higher levels are associated with increased cognitive errors and a decline in performance.^9^ Recognition of the negative impact of higher levels of stress during IHCAs has led to the development of cognitive aids that reduce cognitive load and improve adherence to the American Heart Association's guidelines. Using cognitive aids during IHCAs has been found to decrease stress, improve teamwork, and lead to calmer work environments.^10^ This enabled HCPs to focus more on clinical decision making and patient status, which was associated with better overall patient care. Cognitive aids used during IHCAs may include timers, metronomes, algorithm cards, and smartphone applications.^1^, ^11^, ^12^, ^13^, ^14^

There are many smartphone apps available that offer access to algorithm cards, timers, and metronomes. Unfortunately, very few offer real‐time guidance and audible prompts. Metelmann et al.^13^ found that out of 34 basic life support apps reviewed, only five provided step‐by‐step guidance with accurate American Heart Association information and only one app received an above‐average score on the System Usability Scale (SUS).

### Goals of this investigation

We sought to assess the utility of the Code Blue Leader smartphone application. This app offers both accurate and up‐to‐date ACLS guidelines, while also providing real‐time, step‐by‐step guidance to reduce cognitive load and support HCPs leading IHCAs. It is critically important to evaluate all new cognitive aids in simulated environments as this offers the opportunity to assess safety and efficacy and identify areas of improvement before use in high‐stakes clinical settings.^9^ Therefore, the purpose of this study is to assess the impact of the Code Blue Leader smartphone application on the performance of ACLS‐trained HCPs leading cardiac arrest simulations.

## METHODS

### Study design and setting

This study received approval through a harmonized ethical review between the Island Health Research Ethics Board and the University of Victoria Research Ethics Board. The smartphone application under investigation was created and is owned by the principal investigator (PI) of this study. As such, the PI and other affiliated author were not present in the simulation laboratory during any participant involvement and did not contribute to any data collection or participant assessment. This conflict of interest was disclosed in the application for ethical review. This was an open‐label, randomized controlled trial involving ACLS‐certified medical doctors (MD) and registered nurses (RN). The trial was conducted in the high‐fidelity simulation laboratory situated in the Centre for Interprofessional Clinical Simulation Learning in the Royal Jubilee Hospital in Victoria, BC. A total of seven trial sessions were held from February to April 2021. Because the intervention evaluated was not implemented in a clinical setting, no patients were involved and no health‐related or clinical outcomes were assessed on human participants. Therefore, this study was considered a nonclinical randomized controlled trial and therefore was not preregistered. This trial was not funded or sponsored by any company or institution. No grants or other methods of financial support were utilized.

### Study population

Eligible participants included MDs, RNs, nurse practitioners, licensed practical nurses, advanced care paramedics, and students of these professions who were working, training, or affiliated with the Royal Jubilee Hospital or Victoria General Hospital and held an active ACLS certificate within 2 years of trial participation. The participants were recruited via posters, handout materials, and email recruitment. Only MDs and RNs responded to recruitment efforts. Recruitment was on a first‐come basis until an equal distribution (50% MDs, 50% RNs) was achieved.

### Study protocol

The order of participation was based on order of recruitment. Written, informed consent was obtained from all participants. Prior to randomization, each participant underwent a 10‐min teaching session on how to use the Code Blue Leader smartphone application (version 0.1) based on a standardized checklist of learning points. The Code Blue Leader smartphone application provides real‐time, step‐by‐step guidance and audible prompts for the 2020 ACLS cardiac arrest algorithms. This application was developed for both Apple iOS and Android and is currently undergoing beta testing to prepare for release on both platforms. The Code Blue Leader application will be available to the general population in the near future; however, at the time of writing the manuscript, it was not yet available for download.

**FIGURE 1:**
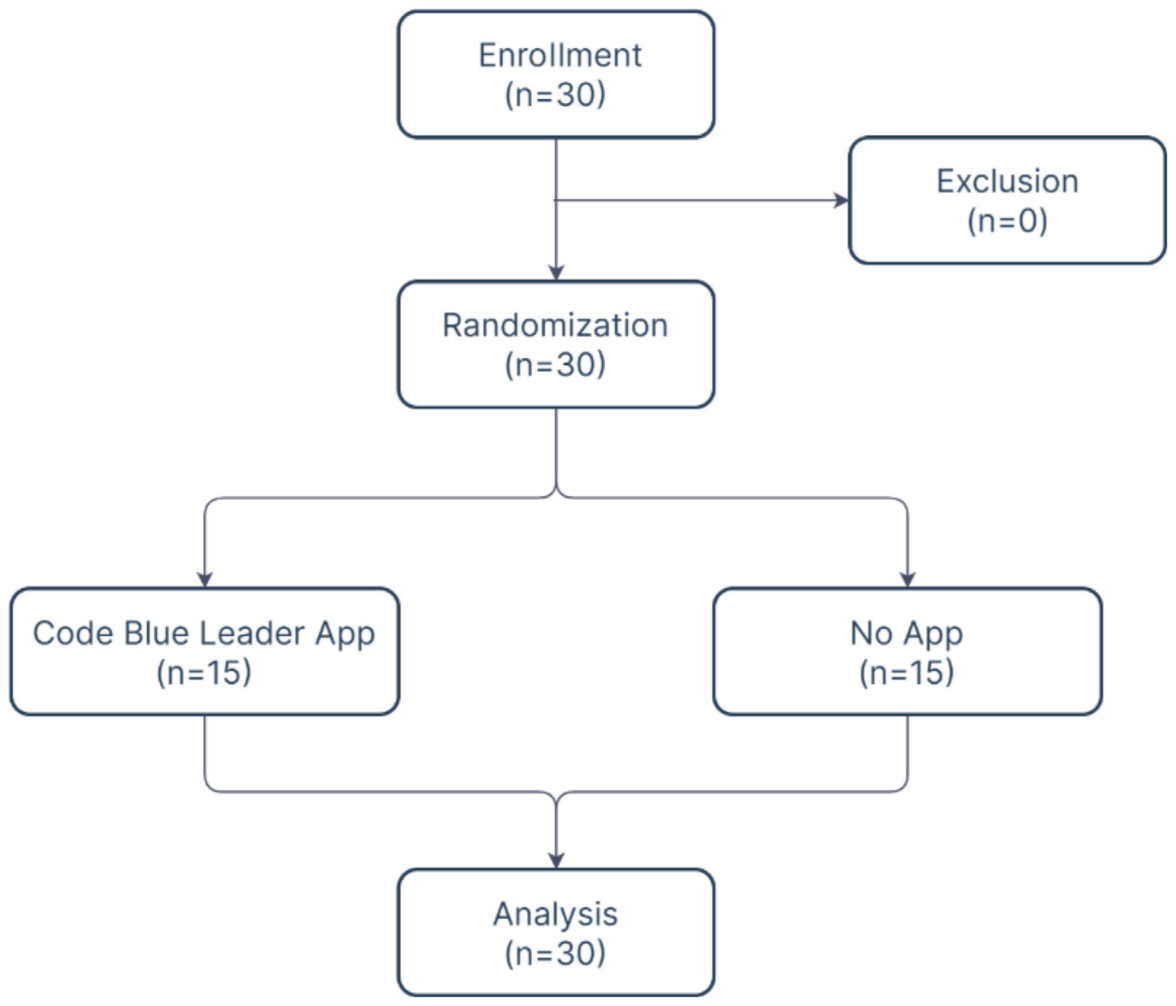
Participant progress through study protocol.

Participants were then randomized via random‐number generator to lead the same high‐fidelity cardiac arrest simulation, either with or without the app. They were stratified based on professional qualification (MD vs. RN) to ensure equal proportions in each study arm (Figure 1). The participants could not be blinded to arm allocation due to the nature of the study. Participants randomized to the app group were given an unlocked smartphone with the Code Blue Leader application preinstalled and were encouraged to use the smartphone app during the ACLS scenario. Those randomized to the control group were told to use any cognitive aids they would normally use to lead the ACLS scenario, including algorithm cards, timers, other smartphone applications, etc.

The simulation lab equipment was set up identically prior to each simulation, based on a standardized checklist. All participants ran the same ACLS scenario in which they responded to a “Code Blue” on an unknown hospital ward. The simulated patient went into cardiac arrest immediately upon participant arrival and sequentially progressed through four pulseless rhythms: ventricular tachycardia, ventricular fibrillation, pulseless electrical activity, and asystole. During the simulation, each participant would lead the scenario independently. They were accompanied by three medically trained assistants in the room who would respond to prompts or follow orders from the participant but would not prompt them or perform any actions independently. Only one trial participant was involved during an individual simulation.

### Outcomes

The primary outcome was the performance score, calculated as the percentage of total correct actions performed. Secondary outcomes included the percentage of critical outcomes performed, the number of incorrect actions, and the chest compression fraction, calculated as the percentage of time spent performing chest compressions. The primary outcome as well as two of the secondary outcomes, percentage of critical outcomes performed and the number of incorrect actions, were assessed based on data collected by a rater using a validated ACLS scoring system. The scoring system used is available as supplemental material accompanying the online article (Figure S1).^15^ The scoring system detailed both correct and incorrect actions to be performed in each ACLS algorithm tested and delineated which of these actions were considered “critical.” As this checklist was validated prior to the most recent ACLS guidelines, a single criterion (the use of atropine) in the “correct actions” category was removed to stay up to date with the current guidelines. The rater was ACLS certified, trained how to use this checklist, and blinded to the study outcomes. Inter‐rater reliability was assessed between the trial rater and an external rater for five practice scenarios prior to the start of the trial, yielding a kappa coefficient of 0.95, which is considered an excellent inter‐rater reliability. A SimMan 3G simulator was used to run the preprogrammed scenario described above and to collect objective data for the final secondary outcome, chest compression fraction.

Following the simulation, to determine usability of the smartphone app, a validated SUS^16^ was filled out by participants randomized to the app group. The overall SUS score is calculated by converting each question's score to a new number, adding them together, then multiplying by 2.5 to convert the original scores of 0–40 to 0–100.

### Data analysis

A target sample size of 30 participants was calculated to detect an effect size of 20% at the 0.05 alpha level with 90% power. The likely performance of the control group (mean ± SD performance score of 66.2% ± 10.4%) was estimated using data from the validation study for the ACLS checklist used in this trial.^15^ Results were analyzed using STATA 15.1. Shapiro–Wilk test for normality, which indicated a nonnormal distribution of the primary outcome data for the app group. Therefore, continuous data are described using median (interquartile range) and comparison for outcomes between groups was accomplished using the Wilcoxon rank‐sum test adhering to intention‐to‐treat analysis.

## RESULTS

### Characteristics of study participants

A total of 15 MDs and 15 RNs were included, and all participants completed the simulation. Between three and five participants ran through the simulation per session. The app group and the control group had similar baseline characteristics (Table 1). Among the MDs, a variety of specialties were represented including family medicine, internal medicine, emergency medicine, anesthesia, radiology, and radiation oncology.

**TABLE 1:** Baseline characteristics for study participants.

	Smartphone App	No App
Age (years)	28 (26–30)	30 (27–39)
Sex, female (%)	10 (66.7)	8 (53.3)
Months since ACLS certification	10 (5–20)	12 (9–18)
Professional qualification		
MD (%)	8 (53.3)	7 (46.7)
RN (%)	7 (46.7)	8 (53.3)
MD specialty		
Family practice (%)	4 (50.0)	3 (42.9)
Internal medicine (%)	2 (25.0)	1 (14.3)
Emergency/anesthesia (%)	1 (12.5)	2 (28.6)
Other (%)^a^	1 (12.5)	1 (14.3)
Years since graduation	2 (1–5)	3 (1–7)
Previously participated in Sim (%)	13 (86.7)	14 (93.3)
Previously ran real code blue (%)	5 (33.3)	4 (26.7)

*Note*: Data are expressed as median (interquartile ratio) or frequency (%).

Abbreviations: ACLS, Advanced Cardiac Life Support; MD, medical doctor; RN, registered nurse.

^a^ Other = radiology and radiation oncology.

### Main results

**FIGURE 2:**
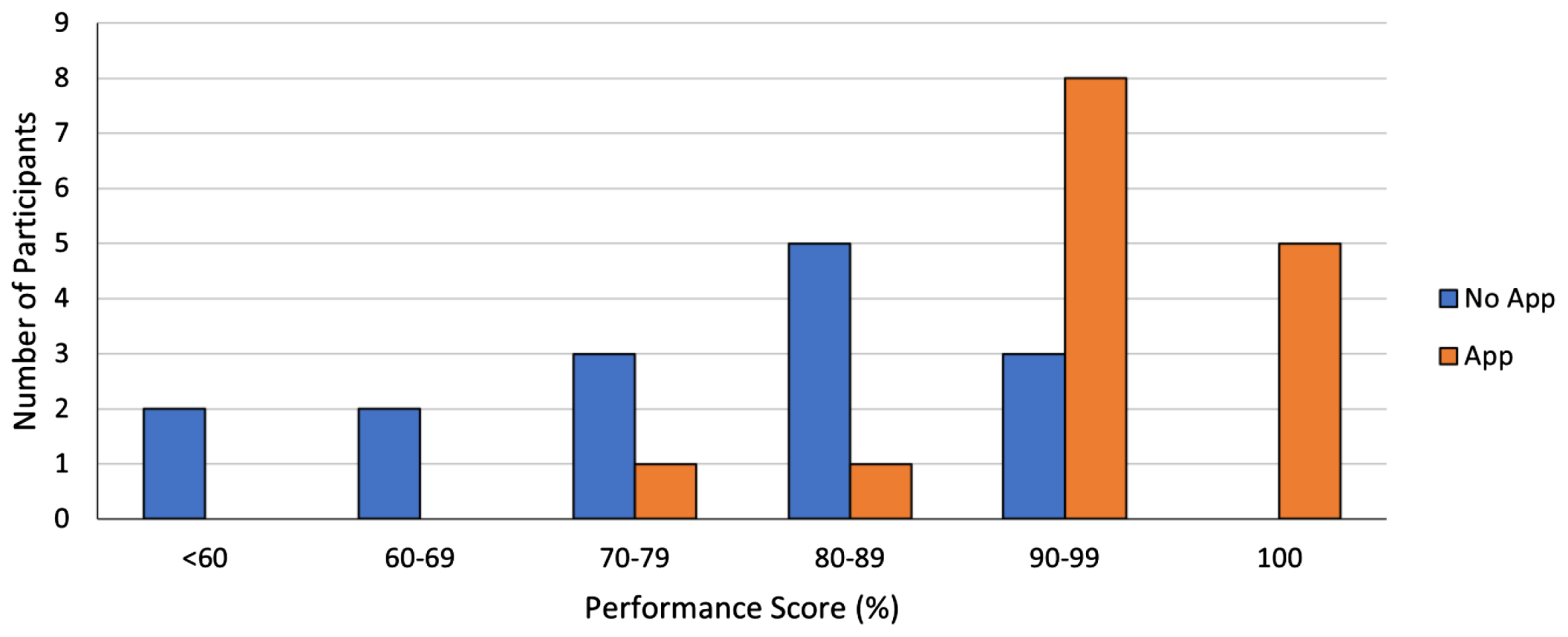
Histogram of ACLS performance scores for participants. ACLS, Advanced Cardiovascular Life Support.

There was a significant difference in the primary outcome (performance scores) between the two groups (Table 2 and Figure 2). The performance score in the app group was 95.3% (93.0%–100.0%) compared to 81.4% (60.5%–88.4%) in the control group, with the Wilcoxon rank‐sum test demonstrating a significant estimated effect size of *r* = 0.69 (*Z* = −3.78, *r* = 0.69, *p* = 0.0002). Prespecified subgroup analysis based on qualification also demonstrates a difference between the intervention and control groups in the primary outcome.

**TABLE 2:** ACLS performance scores (% of correct actions performed) for participants overall and within subgroups based on professional qualification.

	Smartphone App	No App	*p*‐value
Overall	95.3% (93.0%–100.0%)	81.4% (60.5%–88.4%)	0.0002
Subgroups			
MD	96.5% (93.0%–98.8%)	76.7% (60.5%–88.4%)	0.0054
RN	95.3% (93.0%–95.3%)	81% (65.1%–88.4%)	0.0103

*Note*: Data are expressed as median (interquartile ratio).

Abbreviations: ACLS, Advanced Cardiac Life Support; MD, medical doctor; RN, registered nurse.

For the secondary outcomes, Table 3 demonstrates a significant difference between the two groups for the median percentage of critical actions performed, which was 100% (96.2%–100.0%) in the app group compared to 85.0% (74.1%–92.4%) in the control group. The median number of incorrect actions performed was significant with the app group at 1 (1) compared to 4 (3–5) in the control group. The median percentage of time spent performing chest compressions was similar between groups at 75.5% (73.0%–84.0%) in the app group compared to 75.0% (72.0%–85.0%) in the control group.

**TABLE 3:** Percentage of critical actions performed, number of incorrect actions performed, and chest compression fraction for participants.

	Smartphone App	No App	*p*‐value
Percentage of critical actions performed	100% (96.2%–100.0%)	85% (74.1%–92.4%)	0.0001
Number of incorrect actions performed	1 (1–1)	4 (3–5)	<0.0001
Time spent performing chest compressions	75.5% (73.0%–84.0%)	75.0% (72.0%–85.0%)	0.7594

*Note*: Data are expressed as median (interquartile ratio).

The cognitive aids used by the control group included timers, metronomes, and smartphone resources with ACLS algorithms. Unfortunately, the number of participants using each aid and the characteristics of smartphone resources was not collected in further detail.

Table 4 demonstrates the mean scores for each question on the SUS as well as the average overall SUS score. The average SUS score for the Code Blue Leader app was 91. Based on previous research, a SUS score above 68 would be considered above average. Additional results are available as supplemental material accompanying the online article (Table S1).

**TABLE 4:** SUS for participants randomized to use the smart phone app scored from 1 (strongly disagree) to 5 (strongly agree).

Statement	Average Score
I think that I would like to use Code Blue Leader frequently.	4.7
I found Code Blue Leader unnecessarily complex.	1.4
I thought Code Blue Leader was easy to use.	4.7
I think that I would need the support of a technical person to be able to use Code Blue Leader.	1.2
I found the various functions in Code Blue Leader were well integrated.	4.5
I thought there was too much inconsistency in Code Blue Leader.	1.2
I would imagine that most people would learn to use Code Blue Leader very quickly.	4.8
I found Code Blue Leader very cumbersome (awkward) to use.	1.3
I felt very confident using Code Blue Leader.	4.1
I needed to learn a lot of things before I could get going with Code Blue Leader.	1.3
Average overall SUS score = 91^a^	

*Note*: Values are expressed as the mean score for each question.

Abbreviations: SUS, System Usability Score.

^a^ SUS score > 68 is considered above‐average usability.

## DISCUSSION

Use of the Code Blue Leader app significantly improved ACLS performance scores among ACLS‐certified HCPs. This impact was demonstrated among prespecified subgroups (MDs and RNs).

In comparison to the app group, the control group performed significantly less “critical” correct actions while leading cardiac arrest simulations despite still having access to their usual cognitive aids of choice (i.e., timers, metronomes, ACLS algorithms, other smartphone applications). While many cognitive aids have been demonstrated to improve performance when compared to no cognitive aids at all, it is also important to compare different modalities to explore the most efficacious approach. For example, Burden et al.^17^ found that simply providing algorithm cards is not as beneficial as real‐time guidance and prompts. In their study, one group received algorithm cards, while the other group assigned a medical student to read the cards aloud. In the group without the “reader,” 66% of the participants read the cognitive aid silently and then quickly placed it back on the cart; 33% of these participants then picked it up again, briefly read it, and again placed it back on the cart. While reading the algorithm card, participants stopped engaging with the team entirely. The group with an assigned reader performed significantly more critical actions demonstrating the importance of step‐by‐step guidance. Assigning a reader is not always logistically feasible in situations with limited human resources so smartphone applications, such as Code Blue Leader, are a reasonable alternative to fulfill this role.

The control group performed significantly more incorrect actions when compared to the app group. These incorrect actions included, but were not limited to, not identifying the correct cardiac rhythm on the monitor, and administering medications that are not recommended for routine use during ACLS. Spanos and Patterson^18^ discovered that a major barrier for effectively leading an IHCA is accurately identifying the rhythm on the monitor. In their study, half of medical residents identified ventricular fibrillation incorrectly, which led to a significant increase in time to defibrillation. The Code Blue Leader app includes schematics of each rhythm that participants were required to select to designate the ACLS algorithm they were following. This feature was included to support HCPs who lead IHCA infrequently and may benefit from a real‐time visual reminder of each rhythm. Additionally, Benz^3^ reports that using medications inappropriately during IHCAs not only contributes to critical shortages of lifesaving medications but is also associated with decreased rates of ROSC. It should be noted that in the primary characteristics of the study groups, the mean time since completing ACLS certification was less in the app group than the control group (10 months vs. 12 months). It is possible that the difference in recency of the certification may be one of the factors contributing to the differences in correct and incorrect actions between the two groups.

The compression fraction is an important outcome to assess as it is known to be a critical prognostic factor during a resuscitation and previous literature has suggested that the use of cognitive aids may impair the timing of critical interventions, such as initiating CPR. Zanner et al.^19^ assessed the use of a CPR‐guide mobile phone application in a simulated cardiac arrest among final‐year high school students. They found that the time required for action on the unconscious patient for the test group was significantly slower than that in the control group (average time of 4.24 vs. 2.41 min, *p* < 0.001). However, these participants had little or no basic life support training. In our study among HCPs, the compression fraction was nearly identical between the app group and the control group, suggesting that use of cognitive aids among trained individuals does not impede the timing of this critical intervention. Therefore, the app group demonstrated significant improvement in percentage of critical actions and number of incorrect actions as per ACLS, without negatively impacting compression fraction. This is important to note because greater adherence to ACLS guidelines, as demonstrated in the app group compared to the control group in this study, has been associated with increased ROSC and survival to discharge in previous literature.^3^, ^4^, ^5^ While this study was carried out in a simulated environment, previous studies indicate use of the Code Blue Leader application may improve ROSC and survival to discharge in clinical settings as well. However, further research is required to determine whether this is the case.

Resuscitation teams are often multidisciplinary—including MDs, RNs, respiratory therapists, pharmacists, and others who may or may not have ACLS training or experience—and while they are often led by MDs, they may be led by other HCPs with ACLS training. For example, Gilligan et al.^20^ demonstrated that ACLS‐trained emergency department RNs can perform the role of resuscitation team leader as effectively as ACLS‐trained MDs. Additionally, Guetterman et al.^21^ showed that hospitals that encouraged nursing education and nurse‐led interventions in IHCAs had greater rates of IHCA survival than those hospitals that did not. For these reasons, we chose to include ACLS‐trained RNs to test the effectiveness of the Code Blue Leader application in improving resuscitation performance. Though not directly assessed, MDs and RNs demonstrated similar performance scores. This was true for both the control group and the app group. While this was not the focus of our study, this is still an important finding as it contributes to the existing literature that RNs and MDs have comparable levels of competence leading IHCAs. Pallas et al.^22^ found there is significant benefit to nurse‐led codes as this approach reduces cognitive load for MDs, enabling them to focus on other aspects of cardiac arrest management, such as addressing reversible causes.

Müller et al.^23^ used a Kano questionnaire to assess features that both RNs and MDs believe are essential to include in an IHCA phone application. They identified many features that were attractive (i.e., features that are unexpected but lead to user satisfaction when present) and those that were one‐dimensional (i.e., features that are expected and lead to satisfaction when present and dissatisfaction when absent). The Code Blue Leader app includes many of the features that HCPs in the study by Müller et al. identified as attractive or one‐dimensional, which includes rhythm check reminders, resuscitation medication reminders, time recording when an IHCA is called, and a metronome.^23^ These features were included to promote user satisfaction and app usability. In our study, participants in the app group completed the SUS questionnaire, which defines any SUS score greater than 68 as above average usability. The average overall SUS for the Code Blue Leader app was 91 out of 100, which is defined as “best possible” on the SUS adjective rating scale.^24^ In contrast, the mean SUS of several electronic health records used by physicians in the United States was 46.1 out of 100.^25^

The participants in this study demonstrated higher baseline performance scores at 76.0% compared to the study by McEvoy et al.^5^ that validated the use of the scoring system at 66.2%. This difference may be due to the fact that our study only scored participants on the full cardiac arrest algorithms, compared to the validation study that assessed full cardiac arrest in addition to unstable bradycardia and unstable tachycardia ACLS algorithms that add additional layers of complexity. Our findings of improved performance scores reflect the findings of a similar study performed by Low et al.^26^ assessing the use of an algorithm‐based resuscitation app in the United Kingdom called “iResus” that yielded improved results with the use of the app and found that the usability of a smartphone app during resuscitation was deemed to be favorable based on a questionnaire.

The strengths for this trial include that it was well randomized as demonstrated by the evenly distributed baseline characteristics, and it utilized a validated scoring system for the primary outcome and secondary outcomes as well as objective data from the simulation mannequin. Clinically relevant outcomes were assessed including the correct/critical steps required to be performed in a real‐life resuscitation as well as ensuring no detrimental impact on the compression fraction, which is known to be an important prognostic factor. Having limited inclusion criteria increases generalizability to ACLS‐trained MDs and RNs. The simulation was high fidelity in a state‐of‐the‐art simulation lab that allowed for participants to interpret rhythms and assess the patient as they would in a real‐life scenario.

Resuscitation adjuncts providing algorithm, treatment, and timing prompts improve ACLS performance scores. Extrapolation of results to clinical practice is unknown, but some evidence suggests a significant impact of simulation‐based education on quality of care during cardiac arrest in the clinical environment.^27^ Smartphone applications represent an ideal adjunct as they are easy to disseminate and stay up to date with the most current ACLS algorithms, and they offer timed visual and audio prompts that cannot be done using other methods (e.g., ACLS algorithm cards). Finally, they have been shown to be easy to use, even during a cardiac arrest scenario. Future research is required to assess the impact of the Code Blue Leader smartphone application and similar adjuncts on real‐life cardiac arrests and patient‐centered outcomes as well as its utility for other ACLS algorithms, such as unstable bradycardia and tachycardia.

## LIMITATIONS

This study has several limitations. Only the pulseless cardiac arrest algorithm was assessed, so we are unable to speak directly to any impact on other ACLS algorithms such as unstable bradycardia or tachycardia, which may demonstrate different challenges and performance results for participants. For a single participant in the app group, data for compression fraction were not recorded due to a software error in the simulation mannequin. Data for every other outcome (including the primary outcome) were still collected for this individual. Since this was a single isolated event, the missing data were not accounted for in any way.

No blinding was possible as the study arm was visibly apparent to the participants, assistants, and rater. However, the trained rater was blinded to the primary and secondary outcomes of the trial to reduce bias. This trial's inclusion criteria included participants with other professional qualifications including licensed practical nurses and advanced care paramedics. However, it was later discovered that most HCPs with these designations did not have active ACLS certification. The HCPs from these professions who responded to recruitment did not have active ACLS certification and thus did not meet the inclusion criteria required to participate in the study. This, therefore, limits generalizability to MDs and RNs. Finally, whether improved ACLS performance scores lead to improved patient outcomes was not able to be determined through this study.

## CONCLUSIONS

In summary, the Code Blue Leader smartphone app significantly improved the performance of Advanced Cardiovascular Life Support–trained health care professionals in a high‐fidelity cardiac arrest simulation. Use of the app also improved the percentage of critical actions performed, decreased the number of incorrect actions performed, and did not affect the chest compression fraction. Overall, the app was found by participants to have above‐average usability.

## AUTHOR CONTRIBUTIONS

Samuel L. Brophy conceptualized and designed the study, analyzed the results, interpreted analysis of the data, wrote portions of the manuscript, and critically revised the final manuscript and is responsible for the manuscript as a whole. Riley M. Reel and Michael R. McCue collected the data, wrote portions of the manuscript, and critically revised the final manuscript. Tristan D. Jones designed the study and critically revised the final manuscript. Roger D. Dias conceptualized and designed the study, independently reran statistics and interpreted data, and critically revised the final manuscript. Those who contributed to the project but did not write the manuscript are listed in the acknowledgements section.

## CONFLICT OF INTEREST STATEMENT

This trial was not funded or sponsored by any company or institution. No grants or other methods of financial support were utilized. The smartphone application under investigation in this trial was created by the principal investigator (SB) of this study. It is owned by SB and TJ and is currently protected under a provisional patent. As such, SB and TJ were not present in the simulation laboratory during any participant involvement and did not contribute to any data collection or participant assessment. This conflict of interest was disclosed in the application for ethical review. The completion of this trial contributed to SB meeting requirements to complete his master's degree at Harvard University. The other authors declare no conflicts of interest.

## Supporting information


Figure S1

Table S1
