# Supplementary material for: The impact of a smartphone‐based cognitive aid on clinical performance during cardiac arrest simulations: A randomized controlled trial
Source: AEM Educ Train. 2023 Jun 7;7(3):e10880. doi: 10.1002/aet2.10880 (PMC10245296; doi:10.1002/aet2.10880)
Supplement: Supplementary file 1 — Figure S1 [file AET2-7-e10880-s002.pdf]

Figure S1. Correct, incorrect, and critical actions recorded.

Correct Actions	Possible Incorrect Actions
**Assessed rhythm, pulse, & stability**	**Gave any drug prior to shock 2**
Stated diagnosis of ventricular fibrillation	**>3 minutes from rhythm recognition to first shock**
**Started CPR (30:2) until defibrillator attached**	**Gave shock <150J biphasic**
Declared all clear prior to defibrillation	Gave wrong dose of a drug in this pathway
**Immediately defibrillated at 150-200J biphasic after rhythm recognition**	Gave wrong sequence of epinephrine
**Resumed CPR for 5 cycles (30:2)**	Gave wrong sequence of amiodarone/lidocaine
Rhythm & pulse checked (<10 second pause)	Gave atropine
Declared all clear prior to defibrillation	Gave other wrong drug for this pathway
**Defibrillated at 200J biphasic - SHOCK 2**	>1 minute from rhythm recognition to first shock
**Resumed CPR for 5 cycles (30:2)**	>2 minutes from rhythm recognition to first shock
**Gave epinephrine 1mg**	Gave shock >200J biphasic
Rhythm & pulse checked (<10 second pause)	
Declared all clear prior to defibrillation	
**Defibrillated at 200J biphasic - SHOCK 3**	
**Resumed CPR for 5 cycles (30:2)**	
**Gave amiodarone 300mg or lidocaine 1.5mg/Kg IV**	
Considered H's & T's	
Ordered ABG	

**Legend:**
Correct actions
**Critically correct actions**
Incorrect actions
**Critically incorrect actions**

Pulseless Electrical Activity

Correct Actions	Possible Incorrect Actions
**Assessed rhythm, pulse, & stability**	**Administered shock in this pathway**
**Resumed CPR for 5 cycles (30:2)**	Gave amiodarone in this pathway
Stated diagnosis of PEA	Gave lidocaine in this pathway
Rhythm and pulse checked (<10 second pause)	Gave the wrong dose of a drug indicated in this pathway
**Resumed CPR for 5 cycles (30:2)**	Gave adenosine, beta-blockers, or calcium-channel blockers in this pathway
**Gave epinephrine 1mg**	Gave other wrong drug for this pathway
**Considered H’s & T’s and other differential diagnoses**	

Asystole

Correct Actions	Possible Incorrect Actions
**Assessed rhythm, pulse, & stability**	**Administered shock in this pathway**
Confirmed asystole in 2 leads	Gave amiodarone in this pathway
Stated diagnosis of asystole	Gave lidocaine in this pathway
**Resumed CPR for 5 cycles (30:2)**	Gave the wrong dose of a drug indicated in this pathway
**Gave epinephrine 1mg**	Gave adenosine, beta-blockers, or calcium-channel blockers in this pathway
Rhythm and pulse checked (<10 second pause)	Gave other wrong drug for this pathway
**Resumed CPR for 5 cycles (30:2)**	
Rhythm and pulse checked (<10 second pause)	
**Considered H's & T's and other differential diagnoses**	
Ordered ABG	

↓

Assessment of CPR (Every 5 Cycles)

Correct Actions	Possible Incorrect Actions
**Placed backboard under patient**	**CPR not started within 180 seconds of recognizing pulseless state**
**CPR at 30:2 ratio**	CPR not started within 60 seconds of recognizing pulseless state
**Rate of 100-120 compressions/minute verified or corrected if improper**	CPR delayed for >10 seconds at pulse & rhythm check
**Compression depth 1.5-2" verified or corrected if improper**	
**Hand placement: midline, lower half of sternum verified or corrected if improper**	

↓

Airway Management

Correct Actions	Possible Incorrect Actions
**Confirmed chest rise with BMV**	**CPR delayed >60 seconds for ETT placement**
**Requested ETT placement if BMV insufficient**	CPR delayed >15 seconds for ETT placement
Requested ETT placement for secure airway prior to transport, if not already present	CPR delayed >30 seconds for ETT placement

McEvoy MD, Smalley JC, Niertert PJ, et al. Validation of a detailed scoring checklist for use during advanced cardiac life support certification. Simul Healthc. 2012;7(4):222-235. doi:10.1097/SIH.0b013e3182590b07
