# Supplementary material for: The impact of a smartphone‐based cognitive aid on clinical performance during cardiac arrest simulations: A randomized controlled trial
Source: AEM Educ Train. 2023 Jun 7;7(3):e10880. doi: 10.1002/aet2.10880 (PMC10245296; doi:10.1002/aet2.10880)
Supplement: Supplementary file 2 — Table S1 [file AET2-7-e10880-s001.pdf]

Table S1. Number of incorrect actions performed by participants.

Incorrect Action	Number of occurrences
CPR delayed for >10 seconds at pulse & rhythm check	19
Gave wrong sequence of epinephrine	14
Gave any drug prior to shock 2	9
Gave wrong dose of an indicated drug	6
>1 minute from rhythm recognition to first shock	6
Gave other wrong drug for this pathway	4
Shocked patient in PEA pathway	3
Gave wrong sequence of amiodarone/lidocaine	3
Shocked patient in asystole pathway	2
>2 minutes from rhythm recognition to first shock	2
CPR delayed >15 seconds for ETT placement	2
Gave amiodarone in PEA pathway	1
CPR not started within 180 seconds of recognizing pulseless state	1
>3 minutes from rhythm recognition to first shock	1
